# Electroconvulsive Therapy for the Treatment of Protracted Resistant Agitated Psychosis With Comorbid Delirium in the Intensive Care Unit: A Case Report

**DOI:** 10.1155/crps/6633644

**Published:** 2026-09-25

**Authors:** Mohammad Javed, Jack Noto, Allison Trabold, Renzmark Vallesteros, Muhammad Abbas

**Affiliations:** ^1^ Psychiatry, Larkin Community Hospital, South Miami, Florida, USA, larkinhospital.com; ^2^ Psychiatry, Rutgers Health—RWJMS, Piscataway, New Jersey, USA; ^3^ Internal Medicine, Hackensack University Medical Center, Nutley, New Jersey, USA, hackensackumc.org; ^4^ Family Medicine, MyMichigan Medical Center, Midland, Michigan, USA; ^5^ Psychiatry, Hackensack Meridian School of Medicine, Nutley, New Jersey, USA, hmsom.org

**Keywords:** agitated psychosis, case report, ECT, electroconvulsive therapy, ICU delirium, treatment-resistant

## Abstract

Delirium is a common and debilitating condition in the intensive care unit (ICU), leading to prolonged hospital stays, increased mortality, and significant morbidity. While nonpharmacologic interventions are typically the first line of treatment, pharmacologic approaches such as antipsychotics are often used to manage agitation, with variable efficacy. This case report describes a patient with agitated psychosis with comorbid delirium in the ICU, who failed to respond to multiple trials of antipsychotic medications and experienced a prolonged ICU stay. Electroconvulsive therapy (ECT) was initiated, with subsequent improvement in the patient’s symptoms, including the resolution of agitation and delirium. This case underscores the need for early consideration of ECT in treatment‐resistant agitated psychosis with comorbid delirium and highlights the potential benefits of this approach, particularly in patients with complicated or multifactorial presentations.

## 1. Introduction

Delirium is a widely pervasive pathology in hospital settings; this is especially true within the intensive care unit (ICU), where as high as 80% of patients will develop delirium [1]. The development of delirium within this context is associated with prolonged hospital and ICU stays and increased mortality [2]. There are numerous factors which can affect the likelihood of developing delirium within this setting including increasing age, mechanical ventilation, and preexisting cognitive impairment [1]. There are also several environmental factors which make patients within the ICU particularly susceptible to the development of delirium, most notably fragmented sleep [3]. The majority of patients who develop delirium within the ICU typically demonstrate a hypoactive phenotype characterized by psychomotor slowing [1]. Hyperactive and mixed phenotypes, on the other hand, can display psychomotor agitation, which can require the use of restraints and sedation. This can be especially problematic as both of these interventions can lead to a prolongation of delirium [4, 5].

Given the significant morbidity and mortality associated with delirium in the ICU, it is imperative that it is effectively treated. The first‐line intervention for these patients is the initiation of nonpharmacologic interventions which typically include managing pain, reorienting the patient with family figures, and early mobilization [6]. In patients exhibiting agitation, several pharmacologic interventions are typically used to avoid the administration of restraints or delirium‐exacerbating sedatives. Typically, antipsychotics such as haloperidol, olanzapine, and quetiapine have been used to treat agitation in delirium; however, the evidence regarding their efficacy is mixed [4, 7, 8]. Patients who fail to respond to antipsychotic medication for the management of agitated delirium may benefit from electroconvulsive therapy (ECT). There is currently limited literature, however, exploring the use of ECT for the treatment of hyperactive delirium within the ICU setting. A search of PubMed using the terms “ECT,” “Electroconvulsive Therapy,” “Delirium,” and “ICU” yielded limited prospective data, including only one case series of five patients which suggested that ECT may be considered in cases of refractory ICU delirium [9]. Current literature suggests that ECT may modulate neuroinflammatory pathways and neurotransmitter dysregulation implicated in prolonged delirium, though its application in the ICU remains underutilized.

This article describes the case of a patient with agitated psychosis with comorbid delirium within the ICU, who failed to respond to multiple trials of conventional antipsychotic treatments and experienced a protracted stay. The patient was subsequently trialed on ECT and demonstrated resolution of agitation and delirium. Our case draws attention to this difficult‐to‐treat patient population and highlights the need for early consideration of ECT when conventional treatment is not effective.

## 2. Case

The patient is a 47‐year‐old, single, high school‐educated, unemployed man who lives with his sister’s family and his younger son. The patient’s past psychiatric history is notable for three prior psychiatric admissions, the last being 2 years prior; however, the cause of his admissions is unknown. He was brought by emergency services to the trauma bay after being found with a large horizontal neck laceration and a bloody knife nearby. The patient was intubated, connected to a ventilator, transported to the operating room, and underwent emergent surgery for neck exploration and cauterization of the wound, with nasogastric (NG) tube insertion. He was subsequently admitted to the Surgical ICU (SICU). He underwent further surgery 3 days later, where the larynx was successfully repaired, a tracheostomy was performed, and he was connected to a ventilator. The patient was noted to be disoriented to time and place, agitated, and in pain, necessitating the administration of oxycodone 5 mg via NG tube. The patient screened positive for delirium using the Confusion Assessment Method for the ICU (CAM‐ICU). His agitation was quantified using the Richmond Agitation‐Sedation Scale (RASS), which fluctuated between +1 (restless) and +3 (very agitated) prior to sedation. He frequently attempted to pull out his lines, requiring the use of restraints and sedatives. Psychiatry was consulted to assist in management of the patient’s agitated state and over concern for suicidality.

As a result of his tracheostomy, he had difficulty communicating, and collateral history was collected from the patient’s niece and sister. The patient was described by his family as having no friends and being unable to remain employed. He demonstrated paranoid behavior, believing that “people were out to get him” and constantly checked the front door of their house for intruders. The family was unaware of the events leading up to or reason for his prior hospitalizations; however, he was not taking any medications leading up to his current presentation. The family denied hearing the patient express suicidal or homicidal ideation; however, they noted that in the week leading up to his injury, he had begun to eat dinner with the family rather than his usual routine of isolating himself.

After his surgery, the patient remained in the SICU. His course was complicated by ventilator‐associated pneumonia, persistent respiratory failure, and *Clostridioides difficile* infection, leading to a protracted stay in the SICU of 2 months. CT head with and without contrast was unremarkable. The patient remained in acute pain necessitating an increase in dosage and frequency of oxycodone: 10 mg every 4 h at 2 weeks and 15 mg every 4 h at 1 month. He remained delirious and agitated throughout his stay, unable to follow or respond to simple commands and frequently attempted to pull on his lines, requiring the use of restraints and sedation with propofol. During this period, his CAM‐ICU remained persistently positive, and RASS scores consistently necessitated intervention. This presentation was consistent with multifactorial hyperactive delirium. While formal psychometric testing was not feasible due to the patient’s intubated status and agitation, the diagnosis of schizophrenia was established via clinical consensus based on DSM‐5 criteria. Crucially, the family’s collateral history confirmed that paranoid ideation, chronic social isolation, and affective blunting were present well before his admission, allowing the team to differentiate his baseline psychosis from acute ICU delirium. Alternative explanations that were considered include catatonic excitement and delirious mania due to the severity of his psychomotor agitation, which complicated the clinical differentiation between medical delirium and an acute psychiatric exacerbation. The patient was trialed on standing doses of several antipsychotic medications indicated to both reduce agitation and treat his acute psychosis, including quetiapine, 50 mg twice a day; olanzapine, 15 mg three times a day; and paliperidone, 3 mg daily, as well as valproate, 500 mg every 8 h. Haloperidol, 2 mg every 8 h as needed, was additionally used to treat acute agitation. Olanzapine demonstrated some effectiveness in improving the patient’s internal preoccupation, and the patient was continued on a regular dose; however, he continued to be unresponsive to the medical team and his family. An ECT team was consulted to assess suitability for treatment of agitated delirium and negative symptoms.

Due to the patient’s delirium and inability to provide informed consent, the risks and benefits of ECT were discussed with the patient’s authorized surrogate decision‐maker (sister), who provided written informed consent for the procedure. The patient underwent a course of six right unilateral (RUL) ECT treatments. Anesthesia was induced with propofol, and muscle relaxation was achieved with succinylcholine. Seizure quality was monitored via EEG and lower extremity blood pressure cuff, with adequate seizure duration achieved in all sessions. Hemodynamic monitoring was continuous throughout the procedure, with no significant adverse cardiovascular events observed. He demonstrated a measurable reduction in agitation and delirium following the completion of the ECT course. He was able to follow and respond to simple commands, demonstrated an improvement in affective blunting, no longer demonstrated agitated or aggressive behavior, and no longer required the use of sedation or restraints. By the sixth ECT session, propofol, fentanyl, and ketamine infusions had been successfully weaned, although antipsychotic medications were continued. When questioned about his injury, the patient was unable to recall the events leading up to his admission. He was subsequently transferred to an inpatient psychiatric ward and maintained on a regular dose of olanzapine. A comprehensive summary of the patient’s case timeline can be found in the attached table (Table 1).

**Table 1:** Time‐course of admission, clinical events, treatments, and outcomes.

Timepoint	Event
Preadmission (baseline)	Chronic social isolation, unemployment, and paranoid behavior (checking doors for intruders); three prior psychiatric admissions of unknown cause; not on medications. In the week before injury, uncharacteristically began eating meals with family
Day of presentation (day 0)	Found with large horizontal neck laceration and bloody knife nearby; brought in by EMS. Intubated and mechanically ventilated for airway compromise; taken emergently to OR for neck exploration and wound cauterization with NG tube placement; admitted to SICU
Hospital day 3	Return to OR for laryngeal repair and tracheostomy. Disoriented, agitated, and in pain (oxycodone initiated); CAM‐ICU positive, RASS + 1 to +3, pulling at lines requiring restraints. Psychiatry consulted for agitation and suicidality
Week 1	Collateral history obtained (tracheostomy limiting communication); schizophrenia diagnosed by clinical consensus (DSM‐5), differentiated from acute delirium. Standing antipsychotics trialed (quetiapine, olanzapine, paliperidone) plus valproate; haloperidol PRN for agitation
2 weeks	Persistent pain; oxycodone increased to 10 mg every 4 h. Ongoing hyperactive delirium and agitation despite pharmacotherapy
1 month	Oxycodone increased to 15 mg every 4 h. SICU course complicated by ventilator‐associated pneumonia, persistent respiratory failure, and *Clostridioides difficile* infection; propofol sedation continued
Throughout SICU stay (~2 months)	Refractory hyperactive delirium and agitation despite optimized medical management and multiple antipsychotics; olanzapine showed partial benefit; unresponsive to team and family. ECT team consulted; surrogate (sister) provided written informed consent
~2 months	Course of six right unilateral ECT treatments (propofol induction, succinylcholine; EEG/cuff seizure monitoring; no significant cardiovascular events)
Immediately post‐ECT	Marked reduction in agitation and delirium; following simple commands, improved affective range, no longer requiring restraints or sedation; propofol, fentanyl, and ketamine weaned; antipsychotics (olanzapine) continued. Unable to recall events surrounding injury
Post‐ECT/disposition	Transferred to inpatient psychiatric ward, maintained on olanzapine

## 3. Discussion

The case described within our report demonstrates the difficulties in managing and treating patients who develop agitated psychosis with comorbid delirium while in the ICU. It is of little surprise that our patient developed delirium, given his extensive surgery under anesthesia, the nature of his trauma requiring mechanical ventilation, and increasing doses of opioids, factors which were all compounded by his acute psychosis. His delirium was likely further exacerbated by the infections and respiratory failure that developed throughout his stay. Finally, the administration of propofol in order to sedate the patient may have also contributed to the patient’s persistent delirious state. It is important to acknowledge confounding factors in this patient’s recovery. The concurrent treatment of ventilator‐associated pneumonia and the weaning of propofol, a known deliriogenic agent, likely contributed to the clinical improvement of the patient’s medical delirium. However, the persistence of refractory agitation despite these medical corrections suggests that the patient’s primary barrier to recovery was his psychiatric state, which was accompanied by an overlying delirium. The temporal correlation between the initiation of ECT and the rapid resolution of agitation, which had previously persisted despite optimized medical management, suggests a therapeutic role for ECT in this complex presentation.

In the context of a case like this, it is imperative to treat and resolve the patient’s agitation and delirium in order to reduce patient morbidity and mortality, as previously outlined. The patient described within our case did not respond to nonpharmacologic strategies for the attenuation of delirium and did not respond to first‐line pharmacologic interventions for agitation. Notably, quetiapine has previously shown promise for the treatment of agitated delirium among ICU patients who fail to respond to first‐line treatments [10, 11]; however, this response was not seen within our case. Valproic acid has shown similar efficacy in the treatment of refractory hyperactive delirium [12, 13] but failed to demonstrate a meaningful response for our patient.

Given the treatment‐resistant nature of hyperactive delirium within our case, a decision was made to trial ECT. Nielsen et al. [9] previously described five cases of treatment‐resistant hyperactive delirium within the ICU setting, which were successfully treated using ECT. The reports detailed were similar to our case, with patients having failed treatment with antipsychotic medication and having spent at least 2 weeks within the ICU. Notably, however, our patient’s delirious state was likely complicated by his comorbid psychosis, which was distinguished from his acute delirium by his documented preadmission psychiatric history. This comorbid presentation may have affected his response to conventional treatment, limiting comparability with previous literature. To our knowledge, this case is only the second report detailing the use of ECT for the treatment of hyperactive delirium within the ICU setting that has not responded to conventional therapy. Although ECT is a relatively low‐risk procedure, one notable side effect is the development of prolonged post‐ECT delirium, which can arise in 5.7% of patients undergoing ECT [14]. This may be a factor in limiting physician willingness in trialing ECT to treat delirium.

The precise mechanisms underlying the efficacy of ECT in resolving delirium remain under active investigation. Historically, theories relied on anecdotal analogies, such as the hypothesis that ECT‐induced epileptic activity mimics a deeply restorative sleep [3]. However, contemporary models emphasize updated neurobiological and psychophysiological perspectives [15]. Current literature suggests that ECT corrects severe neurotransmitter dysregulation, modulating dopaminergic and cholinergic pathways, and alters cerebral blood flow and neuroanatomical structures to restore neurocognitive homeostasis [15–17]. Furthermore, ECT is thought to mitigate the acute neuroinflammatory cascades that precipitate and sustain critical illness–associated brain dysfunction [15].

Within the case described above, the patient demonstrated a positive response to ECT with regard to negative symptoms; specifically, he regained the ability to interact and speak with the medical team and additionally demonstrated an improvement in affective range. The evidence regarding the use of ECT to treat negative symptoms in schizophrenia is mixed. A review of the existing literature by Zierhut et al. [18] found that 53% of the 35 studies included reported a significant improvement in negative symptoms among patients treated with ECT for schizophrenia. This was particularly true for improving affective blunting among studies which examined symptom subdomains [18, 19]. Despite this, however, Zierhut et al. noted that there were significant variations in study design across projects including cohort size and scales used to assess outcome limiting the generalizability of findings.

## 4. Limitations

There were several limitations within the present article. Most notably, there are multiple features of the case described, namely, the patient’s preexisting psychosis, extensive laryngeal injury, and multiple medical insults during hospitalization, which limit the clinical significance and generalizability of the present study. In addition, the uncertainty regarding the patient’s prior psychiatric admissions and previous psychiatric history likely impacted his current presentation and may further limit the generalizability of the case.

## 5. Conclusion

In summary, the present study adds to the limited literature regarding the use of ECT for the resolution of treatment‐resistant agitated psychosis with comorbid delirium within the ICU. ECT may be an effective treatment in this patient cohort and could be considered at an early stage when possible in order to mitigate extended ICU stays. At present, however, there is limited evidence detailing the effectiveness of ECT within this patient cohort, with the quality of literature consisting solely of case reports/series. Further research, particularly the execution of prospective studies and randomized controlled trials, is needed to assess the effectiveness of ECT compared to conventional treatment within this patient cohort.

## Funding

No funding was received for this research.

## Ethics Statement

This case report was conducted in accordance with the ethical principles outlined in the Declaration of Helsinki. As this is a report of a single case involving anonymized clinical data, formal Institutional Review Board (IRB) approval was waived in accordance with institutional policies at the Hackensack Meridian School of Medicine.

## Consent

Written informed consent was obtained from the patient for the publication of this case report and any accompanying clinical details following the resolution of their delirium and the full restoration of their decision‐making capacity. A copy of the written consent is available for review by the journal’s editorial board or ethics committee upon request.

## Conflicts of Interest

The authors declare no conflicts of interest.

## Data Availability

The data that support the findings of this study are available upon request from the corresponding author, subject to approval by the institutional review board (IRB) and in accordance with the institution’s policies on data sharing. The data are not publicly available due to privacy or ethical restrictions.
